# Featured Organism: *Arabidopsis Thaliana*

**DOI:** 10.1002/cfg.75

**Published:** 2001-04

**Authors:** Jo Wixon

**Affiliations:** Bioinformatics Division HGMP-RC Hinxton, Cambridge CB10 1SA UK

## Abstract

*Arabidopsis* is universally acknowledged as the model for dicotyledonous crop plants. Furthermore, some of the information gleaned from this small plant can be used to aid work on monocotyledonous crops. Here we provide an overview of the current state of knowledge and resources for the study of this important model plant, with comments on future prospects in the field from Professor Pamela Green and Dr Sean May.


**Comparative and Functional Genomics**
*Comp Funct Genom* 2001; **2**: 91–98.
**DOI**: 10.1002/cfg.75

**Featured Organism**

### *Arabidopsis thaliana*

Jo Wixon, Managing Editor
*Bioinformatics Division, HGMP-RC, Hinxton, Cambridge CB10 1SA, UK*

### Abstract

***Arabidopsis*** **is universally acknowledged as the model for dicotyledonous crop plants. Furthermore, some of the information gleaned from this small plant can be used to aid work on monocotyledonous crops. Here we provide an overview of the current state of knowledge and resources for the study of this important model plant, with comments on future prospects in the field from Professor Pamela Green and Dr Sean May. Copyright © 2001 John Wiley & Sons, Ltd.**

### Summary

- **Small flowering plant of mustard family (*Brassicaceae*)**
- **Rapid life cycle, high seed production, easy to cultivate**
- **Small genome, 125 Mb, as five chromosomes**
- **Genome sequence completed, 25,498 genes identified**
- **30% of genes currently have no functional classification**
- **Majority of genome present in two copies, suggesting whole genome duplication**

### Background

*Arabidopsis thaliana* was discovered by Johannes Thal (hence the name *thaliana*) in the Harz mountains in the sixteenth century. Originally, he called it *Pilosella siliquosa*, but several name changes have occurred since then. This plant, commonly known as thale cress, was chosen as a model for scientific research due to the ease with which it can be cultivated (many of these small cress plants can be grown in a small area), its rapid life cycle and high production of seeds. Another feature of this model plant, and a crucial reason in the decision to sequence its genome, was its small genome size, when compared to other plants (Table 1). As a *Brassica*, it is related to oilseed rape (*Brassica napus*) and *Brassica oleracea*, the parent plant from which many years of selective breeding by humans has produced such crops as sprouts, cabbage and cauliflower.

### Tools for study

Reverse genetic methods for gene knockout in *Arabidopsis* include various transposon insertion systems (such as those based upon the maize Ds element) and the *Agrobacterium tumefaciens* transferred DNA (T-DNA). A range of herbicide resistance markers is available, including Basta, and antibiotic resistance markers for kanamycin, hygromycin and streptomycin are also used. The $\beta$-glucuronidase (GUS) reporter gene (or *uidA* gene) has been used to study tissue specific expression patterns in plants since 1987 and is still widely used today. When a promoter-less copy is teamed with the insertion systems, it can be used for promoter or enhancer trap experiments. A T-DNA that contains multimerised transcriptional enhancers can be used to provide over expression (activation) mutants (Weigel *et al.*, 2000). Another T-DNA-based system allows genes neighbouring the insertion

Copyright © 2001 John Wiley & Sons, Ltd.

Table 1. Genome sizes of a range of representative plant species

| Species | | Genome Size |
|---|---|---|
| Brassica | Thale cress | 125 Mb |
| | Oilseed rape | 1200 Mb |
| Legumes | Soya Bean | 1100 Mb |
| | Pea | 4100 Mb |
| Solanaceae | Tomato | 1000 Mb |
| | Potato | 1800 Mb |
| Cereals | Rice | 420 Mb |
| | Wheat | 16000 Mb |

to be expressed under the control of a heat shock promoter (Matsuhara *et al.*, 2000). Many thousands of *Arabidopsis* lines carrying various types of insertions are publicly available from the two recognised stock centres (see Web-based resources).

An alternative approach for repression of gene expression in *Arabidopsis* is the use of antisense expression systems. Sense and antisense silencing approaches have produced somewhat unpredictable results, but recent studies have shown that expression of both sense and antisense RNA together (thus forming double stranded RNA) shows consistent and complete suppression of endogenous genes (Chuang and Meyerowitz, 2000; Levin *et al.*, 2000). Another system, that has been shown to work in a wide variety of plants, is virus induced gene silencing (sometimes referred to as VIGS) in which potato virus X constructs containing transgenes (termed amplicons) have been shown to activate post-transcriptional gene silencing (PTGS) either of stably integrated transgenes or endogenous genes (Dalmay *et al.*, 2000). Work has also been published on a targeted deletion system based upon homologous recombination (Kempin *et al.*, 1997), this is certainly a technique which will be popular in the community if it can be developed to the point at which it has a reasonable efficiency.

Random mutagenesis with ethyl methanesulphonate (EMS) has long been used for forward genetics, but can now be applied to reverse genetics using a new strategy called TILLING (McCallum *et al.*, 2000). Here, the EMS mutagenesis is followed by denaturing high-performance liquid chromatography (DHPLC), to detect base pair changes by heteroduplex analysis, which has been shown to work well in *Arabidopsis* and is fast and automatable.

It has been shown that the *Cre-Lox* system can be used in tobacco to make precise, single insertions, for example of a GUS reporter gene, into lox sites which have previously been introduced into the genome (Day *et al.*, 2000). There may however be problems with the use of the system to insert transgenes, since only half of the insertions analysed in the study showed correct spacial expression of the transgene. Use of this system in *Arabidopsis* with T-DNA vectors containing single lox sites resulted only in a very low frequency of correct insertions and more commonly in chromosomal rearrangements (Vergunst *et al.*, 2000), although it has been used successfully in a mosaic strategy to analyse the role of a flower developmental gene (Sieburth *et al.*, 1998). It is more common though for fate mapping experiments to use transposon excision or X-ray induced chlorophyll sectors.

The completion of the genome sequence of *Arabidopsis* has provided a huge impetus for functional genomics projects. The *Arabidopsis* Functional Genomics Consortium (AFGC, see Web-based Resources) is already providing tools for two approaches towards understanding *Arabidopsis* gene function, a microarray expression analysis service and a service for the identification of T-DNA gene knockout lines. The microarray service is already yielding impressive results (Schaffer *et al.*, 2001). GARNet, the Genomic *Arabidopsis* Resource Network (see Web-based Resources), is a UK initiative for *Arabidopsis* functional genomics, including groups working on Proteomics, Metabolic Profiling, Mutagenesis, Microarrays, Clone Resources and Bioinformatics.

In September 2000, the formation of two EU funded consortia (consisting of researchers working in 10 EU member states) was announced. The EXOn Trapping Insert Consortium (EXOTIC) aims to study the expression patterns of $\sim$5000 *Arabidopsis* genes. The REgulatory Gene Initiative in *Arabidopsis* (REGIA) will investigate the function of almost the entire complement of *Arabidopsis* transcription factors. They aim to identify when they are active, which genes they control and what regulatory networks exist between them. The 2010 project is a new US National Science Foundation (NSF) program aimed at determining the function of all *Arabidopsis* genes by the year 2010. The program calls for funding applications for projects aimed at discovering the functions of networks of genes, or at the development of research tools that

Copyright © 2001 John Wiley & Sons, Ltd.

would allow the community to undertake functional genomics studies.

In proteomics, an approach has been developed which uses *Arabidopsis* callus culture to generate a source of sufficient quantities of organelles to allow for proteomic analysis of mitochondria, endoplasmic reticulum, golgi and plasma membrane (Prime *et al.*, 2000). This will expand the knowledge of organelle composition and protein trafficking in *Arabidopsis*, by determining the subcellular localisation a large number or proteins. A further proteomic study focussing on *Arabidopsis* plasma membrane proteins has shown that they can be grouped into distinct subtypes according to their solubility and electrophoretic properties, which may give clues as to the function of some of these proteins (Santoni *et al.*, 2000).

Metabolic profiling techniques have already been applied to *Arabidopsis*, one obvious driver for this being that plant metabolites are of huge interest to the pharmaceuticals industry. A gas chromatography/mass spectrometry (GC/MS) approach has been used to identify 326 distinct compounds from *A. thaliana* leaf extracts (Fiehn *et al.*, 2000). This study also showed that the metabolic profiles of two *Arabidopsis* ecotypes were different, and that they were more divergent from each other than were single gene mutants of either ecotype from their parent, which supports the idea that this technique can be used for functional genomics studies.

### Current status of genome knowledge

The genome of *Arabidopsis* has been sequenced and was published at the end of last year by a large consortium of laboratories organised into six sequencing groups and a genome analysis group (The *Arabidopsis* Genome Initiative 2000). *Arabidopsis* has five chromosomes, which are all between 17 and 29 Mb in size, giving a genome of ~125 Mb. The sequence covers 115.4 Mb of the genome (only the centromeres and rDNA repeats remain unsequenced). The chromosomes have been shown to all be very similar in terms of base composition (%GC) and gene density. The genome is predicted to contain 25 498 genes. The average *Arabidopsis* gene is ~2 kb long, with five exons, and the average length of peptide encoded is ~430 amino acids. The functions of ~69% of the genes have been predicted based upon homology to genes in other organisms which are of known function, the remaining ~30% are of unknown function. Only 9% of the genes had been characterised experimentally. In contrast to those genes involved in protein synthesis, 48–60% of which have eukaryotic homologues, the genes involved in transcription are more divergent from those of other eukaryotes, with only 18–23% having homologues in *S. cerevisiae*, *C. elegans*, *Drosophila* and human. The metabolism and energy functional categories show quite a high proportion of genes with bacterial homologues. This will partly be due to the high conservation of some of these genes across all species, but others will have been acquired from the ancestor of the plastid (chloroplast).

The proteins can be grouped into 11 601 protein families, a similar number to those observed for *C. elegans* and *Drosophila*. This indicates that this is a sufficient number of protein types to support a variety of multicellular eukaryotic lifestyles. However, a substantially lower proportion of proteins (35%) are unique, and a larger proportion of proteins are in families with five or more members than in *C. elegans* or *Drosophila*, reflecting the marked redundancy in this genome. It should be noted however that this does not necessarily correlate with functional redundancy of these genes. Analysis for conserved protein domains revealed that around 150 of the protein families appear to be unique to plants, several of which are transcription factors.

Analyses of the whole genome sequence revealed 1528 tandem arrays of genes, with 17% of Arabidopsis genes being found in tandem arrays. Several large regions (100 kb or larger) of duplication were found, these account for 60% of the genome. Many of these duplicated regions have often undergone further small-scale rearrangements, evidenced by local gene order changes. The majority of the segments are found in two copies, implying that Arabidopsis could have had a tetraploid ancestor. Polyploidy is common in plant lineages, and is thought to be a key factor in plant evolution. However, the level of conservation of the duplicated segments varies, which could imply that several large duplications occurred independently, rather than one whole genome duplication.

Whilst there is a growing current of opinion that the *Arabidopsis* genome does not share enough synteny with cereal genomes to allow for comparative mapping, it is possible to identify cereal homologues of some *Arabidopsis* genes where functional

Copyright © 2001 John Wiley & Sons, Ltd.

information can be successfully transferred. There are already important examples, such as the *Arabidopsis* gibberellin insensitive gene (GAI), which was shown to be the homologue of reduced height (dwarf) loci in wheat, maize and rice (Peng *et al.*, 1999). Recent studies comparing sequence from a BAC clone from tomato (Ku *et al.*, 2000), and chosen sequence segments from three soybean linkage groups (Grant *et al.*, 2000), to the near-complete genome sequence of *Arabidopsis* have shown that there is significant synteny between these species and *Arabidopsis*, indicating that, for these more closely related species, it will be possible to use comparative mapping with *Arabidopsis*. Both studies also indicate that large-scale genome duplication events, or perhaps even a whole genome duplication event, have occurred during the evolution of the *Arabidopsis* genome.

### Future aims

Professor Pamela Green is at the Plant Research Laboratory of Michigan State University, she is the PI of the AFGC (see Web-based Resources). Her group studies regulation of gene expression in *Arabidopsis* at the level of mRNA degradation. She expressed enthusiasm that the completion of the *Arabidopsis* genome sequence will greatly enhance our ability to address gene function in plants. She points out that the *Arabidopsis* community now know how many genes are in gene families, which genes from other organisms are absent or present, and are gaining insight as to how the genome evolved from the study of natural variation. Numerous new putative genes have been identified on the basis of their predicted open reading frames. Some of the direct advantages that she can see coming from this knowledge are that it will enhance the ability to design more comprehensive DNA microarrays and gene chips for gene expression profiling, and more comprehensive gene knock out strategies. Her view is that, for the power of expression profiling to be realized (such as for the identification of regulatory networks), the data must be publicly available, ideally though a common repository such as TAIR (see Web-based Resources).

As the efforts to determine the flanking sequences of T-DNA insertional mutations are scaled up, she sees the current annotation of the sequence becoming an invaluable tool to pinpoint those genes that have been disrupted. However, her feeling is that the whole *Arabidopsis* community must contribute to the future development and enhancement of these resources. For this, Prof. Green sees a need for large and small-scale investigations to contribute to updating and enhancing the annotation. One important step in this will be to identify full-length cDNAs to accurately delineate the transcribed regions, which are often difficult to predict precisely using computational tools. She further notes that genes such as novel non-coding RNA genes or genes encoding small peptides may have been largely missed because of their small size and lack of a long ORF. Nevertheless, as the community strives to develop strategies to meet these and other exciting challenges, she knows that many share her sentiment that it's never been more fun to work with *Arabidopsis*.

Dr Sean May is Director of the Nottingham *Arabidopsis* Stock Centre (NASC), PI of the *Arabidopsis* Genome Resource (AGR) for UKCropNet and an active member of GARNet (see Web-based resources). In his view, the publication of the full sequence of *Arabidopsis* has permanently changed how almost every aspect of biological research on *Arabidopsis* (and indeed many other plants) will be done. It is now possible to fix the position of any unambiguous gene sequence onto the genome map and as a consequence, he feels that gene discovery is now arguably the province of bioinformatics, rather than that of the molecular biologist. He pointed out that the community now appreciate how many genes (and gene families) it takes to make a fully functioning plant. The large number of gene duplications (more than most people expected) suggests that *Arabidopsis* may not be quite the 'minimal' plant that was originally anticipated, but he suggests that this may just make it a better model with regard to gene redundancy and mechanisms for allele suppression. He appreciates the value of comparing the public and private *Arabidopsis* sequences, which has given access to every single phenotypic difference between Columbia and Landsberg at a genetic level.

For the first time, sequences derived from insert flanking regions (transposon or otherwise) can be unambiguously positioned onto the genome and directly correlated with an observed or derived phenotype. At the stock centre, he expects to see a large proportion of the nearly 200 000 unannotated

Copyright © 2001 John Wiley & Sons, Ltd. 

insert lines now available being precisely located in defined genes. In 2–3 years he anticipates having a homozygous or heterozygous insert mutant for almost every single gene in *Arabidopsis*. He also commented that the UKCropNet resource, AGR, holds the entire *Arabidopsis* Genome Initiative sequence linked by BLAST analysis to all other plant ESTs and all public *Arabidopsis* insert flanking sequences. *Arabidopsis* is already the acknowledged model for dicotyledonous crop species, so this resource will allow researchers to find insert 'knockouts' in *Arabidopsis* genes that match their favourite crop genes. In addition, he sees the entire genome sequence facilitating the identification of gene control elements. These could be clustered for similarity and cloned *en mass* for expression analysis or for ectopic transgene expression.

He feels that gene analysis no longer needs to be driven by obvious phenotypes or blind mutational analysis. Given appropriate annotation, and using PCR primers derived from the sequence, it will shortly be possible to make a complete *Arabidopsis* transcriptome microarray. Such resources will provide a simultaneous and unbiased assessment of thousands of identified genes, changing the focus of experiments towards the analysis of patterns of genes and the interactions between these genes. The days of the 'one gene at a time' style of analysis may be numbered once the complexity of gene interactions is made easily accessible to the community.

Possible candidate genes for quantitative traits (QTLs) should also be easier to infer on the full sequence, he thinks. In order to pinpoint the correct contributory locus for a QTL, he suggests that genes could even be complemented with 'Binary vector BACs' chosen from a defined region on the genome.

In conclusion, he said, "We are all involved in doing the 25,000-gene-piece *Arabidopsis* jigsaw. We have now done the edge and bits of the sky, but the rest seems to be just a matter of slotting the pieces into the only places where they fit and then standing back to look at the big picture".

### Web-based resources

### General Information and Links

The *Arabidopsis* Information Resource (TAIR)
**http://www.arabidopsis.org/home.html**
This site has general information, databases and tools for *Arabidopsis* researchers (see also: Huala *et al.*, 2001). It also provides access to the SNPs and InDels identified by workers at Cereon by comparing their *Landsberg erecta* ecotype data with the public project *Columbia* ecotype data, at: **http://www.arabidopsis.org/Cereon/index.html**

Arabidopsis Links (Arabinet)
**http://weeds.mgh.harvard.edu/atlinks.html**
This site provides a categorised list of *Arabidopsis* links, with brief descriptions of each site.

Lehle Seeds home page
**http://www.arabidopsis.com/**
This site reports a wide range of *Arabidopsis* news items, and lists relevant conferences, publications, laboratories and patents. The site also provides access to the Lehle Seeds *Arabidopsis* catalogue and the rest of their site.

### Databases

MIPS *Arabidopsis thaliana* database (MATDB)
**http://websvr.mips.biochem.mpg.de/proj/thal/**
This database contains all the data produced by the AGI. It has lists of features found in the genome, functional catalogues for each chromosome, protein structure information, some comparative genomics data, and maps and other graphical representations of the chromosomes.

The TIGR *Arabidopsis thaliana* Database
**http://www.tigr.org/tdb/at/at.html**
This site is home to the *A. thaliana* annotation database, which, when complete, will hold all the AGI data, annotated to a uniform standard. There is also a Landsberg erecta random sequence database, which will be used to detect SNPs and InDels, and the *A. thaliana* gene index (AtGI), which contains all publicly available *A. thaliana* EST and transcript entries and any contigs that can be built from them.

Cold Spring Harbor Labs Arabidopsis Sequencing
**http://nucleus.cshl.org/protarab/**
This site provides access to the sequence data generated at CSHL. There is also a database of repeated sequences found in the Arabidopsis genome, a gene name search tool and information on their annotation tools and strategy.

Copyright © 2001 John Wiley & Sons, Ltd.

The Kazusa *Arabidopsis* data opening site (KAOS)
**http://www.kazusa.or.jp/kaos/**
This site has a clickable diagram of the chromosomes which links through to tables of clones in each region and links to their database entries. TBLASTN, BLASTN and keyword searches of the data are also offered.

Genoscope *Arabidopsis* Project
**http://www.genoscope.cns.fr/externe/English/Projets/Projet_A/organisme_A.html**
The Genoscope team co-ordinated the sequencing of the bottom arm of chromosome III and participated in the BAC end sequencing, this site provides graphical displays and BLAST searches of their data.

Database of *Arabidopsis thaliana Annotation* (DAtA)
**http://luggagefast.stanford.edu/group/arabprotein/**
This resource contains information on predicted coding sequences and on protein motifs and protein similarities detected for the proteins predicted from the genome sequence.

PathDB – Metabolic Pathways Database
**http://www.ncgr.org/software/pathdb/**
This database includes reconstructions of *Arabidopsis* metabolic pathways built from the genome sequence, by taking all the predicted enzymes. This covers mainly primary metabolism.

UK CropNet (UK Crop Plant Bioinformatics Network)
**http://ukcrop.net**
This site hosts a wide range of databases and software for crop plant research, including the *Arabidopsis* Genome Resource (AGR).

Demeter's Genomes
**http://ars-genome.cornell.edu/**
This site has a huge collection of mainly plant genome databases, and includes a mirror of AGR.

### Genomics Resources

CBC: Arabidopsis cDNA Sequence Analysis Project
**http://www.cbc.umn.edu/ResearchProjects/Arabidopsis/**
This is a joint project between the University of Minnesota and Michigan State University. The MSU researchers are planning to generate partial 5′ sequence from ~36,000 Arabidopsis cDNAs from a normalised library generated from a mixture of sources (roots, leaves and seedlings). The Minnesota group are involved in automating the data acquisition, creating the database and generating and using tools for data mining.

Meinke Lab Home Page
**http://mutant.lse.okstate.edu/**
This site is a store of information on nomenclature, mutant gene symbols, linkage data and genetic maps of mutant genes. There is also a table of email addresses for the laboratories that have contributed linkage data and forms for the submission of data on new mutations.

### Functional Genomics Projects

Arabidopsis Functional Genomics Consortium (AGFC)
**http://afgc.stanford.edu/**
This project is providing a microarray expression analysis service and a resource of T-DNA gene knockout lines. The Stanford site provides access to information on the microarray project and guides, protocols and instructions for applying to use the service. The site also supports a mailing list for the discussion of microarray technologies and their applications in plant biology (**http://genome-www.stanford.edu/email/plantarrays.html**). The knockout facility page (**http://www.biotech.wisc.edu/Arabidopsis/**) is hosted by the University of Wisconsin. All the information required to use the service to identify a mutant of interest from their collection can be found there.

Genomic *Arabidopsis* Resource Network (GARNet)
**http://garnet.arabidopsis.org.uk**
A UK initiative for functional genomics of *Arabidopsis*, including Proteomics, Metabolic Profiling, Mutagenesis, Microarray, Clone Resource and Bioinformatics projects.

Arabidopsis Transposon Insertion Service
**http://www.jic.bbsrc.ac.uk/STAFF/michael-bevan/ATIS/**
This project to provide sequence data on the sites of transposon insertions is part of GARNet. The team aim to provide insertion site sequence information from lines derived from three populations, which between them give loss-of-function, gain-of-function and expression pattern monitoring information.

Cold Spring Harbor *Arabidopsis* Genetrap Database
**http://spot.cshl.org/genetrap_database/mainframe.html**
This database holds records on a collection of transposon insertion lines that have a unique insertion of a genetrap (GT) or enhancer trap (ET) transposable Ds element. The lines have been stained for reporter gene expression in the seedling and the site of insertion of some of the lines has been sequenced.

Copyright © 2001 John Wiley & Sons, Ltd.

<u>T-DNA enhancer traps in Arabidopsis</u>
**http://www.dartmouth.edu/~tjack/**
This site provides data on the GUS staining patterns obtained from a large population of enhancer trap lines. These lines are available from both stock centres (see below).

### Stock centres

<u>Arabidopsis Biological Resource Centre (ABRC)</u>
**http://www.biosci.ohio-state.edu/~plantbio/Facilities/abrc/ABRCHOME.HTM**
The ABRC collects, preserves and distributes seeds, DNA clones and libraries to researchers from North America. The centre holds the large insert clones used for the sequencing project, T-DNA mutagenised populations and over 40,000 EST clones.

<u>Nottingham Arabidopsis Stock Centre (NASC)</u>
**http://arabidopsis.org.uk/**
The NASC collects, preserves and distributes seeds stocks, DNA, macro-filters and microarrays for Arabidopsis worldwide but principally to European researchers. Seed stocks include mutants, mapping lines and insertion lines. NASC is also home to AGR (see Databases) and InsertWatch – the public insert sequence database.

<u>*Arabidopsis* Information Management System (AIMS)</u>
**http://aims.cps.msu.edu/aims/**
AIMS supports the database needs of the ABRC. Users can search the catalogues and place orders from the ABRC at this site.

### Arabidopsis books

### General

Mary Anderson and Jeremy Roberts (eds). 1998. *Arabidopsis*: *Annual Plant Reviews*, *Vol.1*. CRC Press, Boca Raton, FL, USA.
Elliot M. Meyerowitz, Chris R. Somerville (eds). 1994. *Arabidopsis*. CSHL Press, New York, USA.
John L. Bowman (ed). 1993. *Arabidopsis*: *an Atlas of Morphology and Development*. Springer-Verlag, Berlin & New York.

### Protocols and methods

Csaba Koncz, Nam-Hai Chua, Jeff Schell (eds). 1992. *Methods in Arabidopsis research*. World Scientific Publishing.
J.M. Martinez-Zapater, J. Salinas (eds). 1998. *Arabidopsis Protocols 82*. Humana Press.
Zoe Wilson (ed). 2000. *Arabidopsis*: *A Practical Approach*. Oxford University Press, Oxford, UK.

### References

Chuang CF, Meyerowitz EM. 2000. Specific and heritable genetic interference by double-stranded RNA in *Arabidopsis thaliana*. *Proc Natl Acad Sci U S A* **97**: 4985–4990.

Dalmay T, Hamilton A, Mueller E, Baulcombe DC. 2000. Potato virus X amplicons in *Arabidopsis* mediate genetic and epigenetic gene silencing. *Plant Cell* **12**: 369–379.

Day CD, Lee E, Kobayashi T, *et al.* 2000. Transgene integration into the same chromosome location can produce alleles that express at a predictable level, or alleles that are differentially silenced. *Gene Dev* **14**: 2869–2880.

Fiehn O, Kopka J, Dormann P, Altmann T, Trethewey RN, Willmitzer L. 2000. Metabolite profiling for plant functional genomics. *Nature Biotech* **18**: 1157–1161.

Grant D, Cregan P, Shoemaker RC. 2000. Genome organization in dicots: Genome duplication in *Arabidopsis* and synteny between soybean and *Arabidopsis*. *Proc Natl Acad Sci U S A* **97**: 4168–4173.

Huala E, Dickerman AW, Garcia-Hernandez M, *et al.* 2001. The *Arabidopsis* Information Resource (TAIR): a comprehensive database and web-based information retrieval, analysis, and visualization system for a model plant. *Nucleic Acids Res* **29**: 102–105.

Kempin SA, Liljegren SJ, Block LM, Rounsley SD, Yanofsky MF, Lam E. 1997. Targeted disruption in *Arabidopsis*. *Nature* **389**: 802–803.

Ku HM, Vision T, Liu JP, *et al.* 2000. Comparing sequenced segments of the tomato and Arabidopsis genomes: Large-scale duplication followed by selective gene loss creates a network of synteny. *Pro Natl Acad Sci U S A* **97**: 9121–9126.

Levin JZ, de Framond AJ, Tuttle A, Bauer MW, Heifetz PB. 2000. Methods of double-stranded RNA-mediated gene inactivation in *Arabidopsis* and their use to define an essential gene in methionine biosynthesis. *Plant Mol Biol* **44**: 759–775.

Matsuhara S, Jingu F, Takahashi T, Komeda Y. 2000. Heat-shock tagging: a simple method for expression and isolation of plant genome DNA flanked by T-DNA insertions. *Plant Journal* **22**: 79–86.

McCallum CM, Comai L, Greene EA, Henikoff S. 2000. Targeted screening for induced mutations. *Nature Biotech* **18**: 455–457.

Peng J, Richards DE, Hartley NM, *et al.* 1999. 'Green revolution' genes encode mutant gibberellin response modulators. *Nature* **400**: 256–261.

Prime TA, Sherrier DJ, Mahon P, Packman LC, Dupree P. 2000. A proteomic analysis of organelles from *Arabidopsis thaliana*. *Electrophoresis* **21**: 3488–3499.

Santoni V, Kieffer S, Desclaux D, *et al.* 2000. Membrane proteomics: Use of additive main effects with multiplicative interaction model to classify plasma membrane proteins according to their solubility and electrophoretic properties. *Electrophoresis* **21**: 3329–3344.

Schaffer R, Landgraf J, Accerbi M, Simon V, Larson M, Wisman

Copyright © 2001 John Wiley & Sons, Ltd. 

E. 2001. Microarray Analysis of Diurnal and Circadian-Regulated Genes in *Arabidopsis*. *Plant Cell* **13**: 113–123.

Sieburth LE, Drews GN, Meyerowitz EM. 1998. Non-autonomy of AGAMOUS function in flower development: use of a Cre/loxP method for mosaic analysis in Arabidopsis. *Development* **125**: 4303–4312.

The *Arabidopsis* Genome Initiative. 2000. Analysis of the genome sequence of the flowering plant *Arabidopsis thaliana*. *Nature* **408**: 796–815.

Vergunst AC, Jansen LET, Fransz PF, de Jong JH, Hooykaas PJJ. 2000. Cre/lox-mediated recombination in Arabidopsis: evidence for transmission of a translocation and a deletion event. *Chromosoma* **109**: 287–297.

Weigel D, Ahn JH, Blazquez MA, *et al.* 2000. Activation tagging in *Arabidopsis*. *Plant Physiology* **122**: 1003–1013.

---

*Comparative and Functional Genomics* is a cross-organism journal, publishing studies on complex and model organisms. The 'Featured Organism Article' aims to present an overview of an organism, primarily for those working on other systems. It provides background information on the organism itself and on genomics studies currently in progress, it also gives a list of web sites containing further information and a summary of the status of the study of the genome. These sections are a personal critical analysis of the current studies of the particular organism. The 'Future Aims' section is intended to be of interest to readers who work on the chosen organism and those who study other systems, and the opinions expressed therein are those of the named contributors.

Many thanks to Professor Ottoline Leyser (Section Editor – *Arabidopsis*) for her critical appraisal of this article and to Professor Pam Green and Dr Sean May for sharing their thoughts on the future of *Arabidopsis* genomics research with me.

The
Genomics
Website at Wiley www.wiley.co.uk/genomics

The **Genomics** website at Wiley is a **DYNAMIC** resource for the genomics community, offering **FREE** special feature articles and new information **EACH MONTH**.

Find out more about *Comparative and Functional Genomics*, and *Proteomics*, and how to view many articles **FREE OF CHARGE**!

Visit the **Library** for hot books in Genomics, Bioinformatics, Molecular Genetics and more.

Click on **Journals** for information on all our up-to-the minute journals, including: *Genesis, Bioessays, Gene Function and Disease, Human Mutation, Genes, Chromosomes and Cancer* and the *Journal of Gene Medicine.*

Let the **Genomics** website at Wiley be your **guide** to genomics-related web sites, manufacturers and suppliers, and a calendar of conferences.

WILEY

2171

Copyright © 2001 John Wiley & Sons, Ltd. 
